# *TERT* de novo mutation-associated dyskeratosis congenita and porto-sinusoidal vascular disease: a case report

**DOI:** 10.1186/s13256-025-05031-6

**Published:** 2025-01-23

**Authors:** Ge Yu, Guijie Xin, Xu Liu, Wanyu Li, Chen Shao, Runping Gao

**Affiliations:** 1 Department of Hepatic Biliary Pancreatic Medicine, First Hospital of Jilin University, 1 Xinmin Avenue, Changchun, 130021 China; 2 Department of Infectious Diseases, First Hospital of Jilin University, Changchun, China; 3 Department of Pathology, Beijing YouAn Hospital, Capital Medical University, Beijing, China

**Keywords:** Dyskeratosis congenita, Porto-sinusoidal vascular disease, Telomerase reverse transcriptase, Mutation, Telomere biology disorder

## Abstract

**Background:**

Dyskeratosis congenita is a rare genetic disease due to telomere biology disorder and characterized by heterogeneous clinical manifestations and severe complications. “Porto-sinusoidal vascular disease” has been recently proposed, according to new diagnostic criteria, to replace the term “idiopathic non-cirrhotic portal hypertension.” TERT plays an important role in telomeric DNA repair and replication. A *TERT* c.2286 + 1G/A mutation in a splicing consensus site was identified in a patient with pulmonary fibrosis. Recently, a pathogenic de novo *TERT* c.280A > T variant was associated with diffuse lung disease in an infant.

**Case presentation:**

A 16-year-old Han male patient experienced unexplained black stool for 7 days, accompanied by dizziness and fatigue. On examination, there were mesh pigmentations on the exposed areas of the skin on both hands and feet. Laboratory testing revealed moderate hemorrhagic anemia and mild elevation of alanine aminotransferase. A computed tomography scan showed portal hypertension, esophageal and gastric varices, and splenomegaly. The liver stiffness measurement by FibroScan was 6.0 kPa. Liver biopsy revealed typical features of porto-sinusoidal vascular disease. Whole exome sequencing identified a heterozygous *TERT* c.2286 + 1G > A de novo mutation and quantitative polymerase chain reaction revealed very short telomeres (less than the first percentile for his age). The patient was diagnosed as *TERT* de novo mutation-related dyskeratosis congenita and porto-sinusoidal vascular disease. He underwent esophageal and gastric variceal ligation treatment and received a carvedilol tablet (12.5 mg) every morning. After 6 months, he has moderate iron deficiency anemia and has started receiving polysaccharide iron complex therapy.

**Conclusion:**

When discovering reticular rash and unknown portal hypertension, it is necessary to perform whole exome sequencing and chromosome length testing to clarify the possibility of dyskeratosis congenita/telomere biology disorder with porto-sinusoidal vascular disease.

## Introduction

Dyskeratosis congenita (DC) is a rare genetic disease due to telomere biology disorder (TBD) and exhibits heterogeneous clinical features, varying from atypical forms to the classical triad of leukoplakia, nail dystrophy, and reticular pigmentation, as well as those with severe complications, such as bone marrow failure, immunodeficiency, cancer predisposition, pulmonary fibrosis, liver fibrosis, and portal hypertension [1–4].

Porto-sinusoidal vascular disease (PSVD) has been recently proposed, according to new diagnostic criteria, to replace the term idiopathic non-cirrhotic portal hypertension. The diagnosis of PSVD is based on liver biopsy and the absence of cirrhosis with or without signs of portal hypertension or histological lesions involving the portal venules or sinusoids [5, 6]. The onset of PSVD is related to chronic exposure to various drugs and toxins, and to several systemic conditions including thrombophilia, hematologic disease, gut disease, autoimmune disease, immunodeficiency, and genetic disorders [7].

Telomeres, the protein–DNA complexes located at the ends of chromosomes, are essential for maintaining genomic integrity and cell division cycles. Thus far, pathogenic or likely pathogenic germline variants in 19 genes, including *TINF2*, *TERC*, *TERT*, and others, have been reported to cause TBD [8]. Variants in these genes can be inherited in either X-linked recessive, autosomal dominant, or autosomal recessive forms, and can also occur de novo [2, 9, 10]. Telomerase reverse transcriptase (TERT) plays an important role in telomeric DNA repair and replication. TERT mutations may also lead to aplastic anemia, and pulmonary and hepatic fibrosis in DC without skin and nail changes [11, 12]. A *TERT* c.2286 + 1G/A mutation in a splicing consensus site was identified in a patient with pulmonary fibrosis [13]. Recently, a pathogenic de novo *TERT* c.280A > T variant was associated with diffuse lung disease in an infant [14]. However, TERT p.P632R and p.T726M mutations have not been found to have an impact on the telomerase activities in patients with DC [11]. Recently, c.2707A > G or c.1663G > A variants in the *TERT* gene in two patients with DC (in the heterozygous state) have been detected with uncertain clinical significance [15]. Therefore, the identification of pathogenic variants in the *TERT* gene in patients with DC faces significant challenges and requires great attention [16, 17].

In this report, we describe a rare case of *TERT* c.2286 + 1G/A de novo mutation-related DC accompanied with PSVD. This rare clinical condition has never been reported in literature.

## Case presentation

**Fig. 1:**
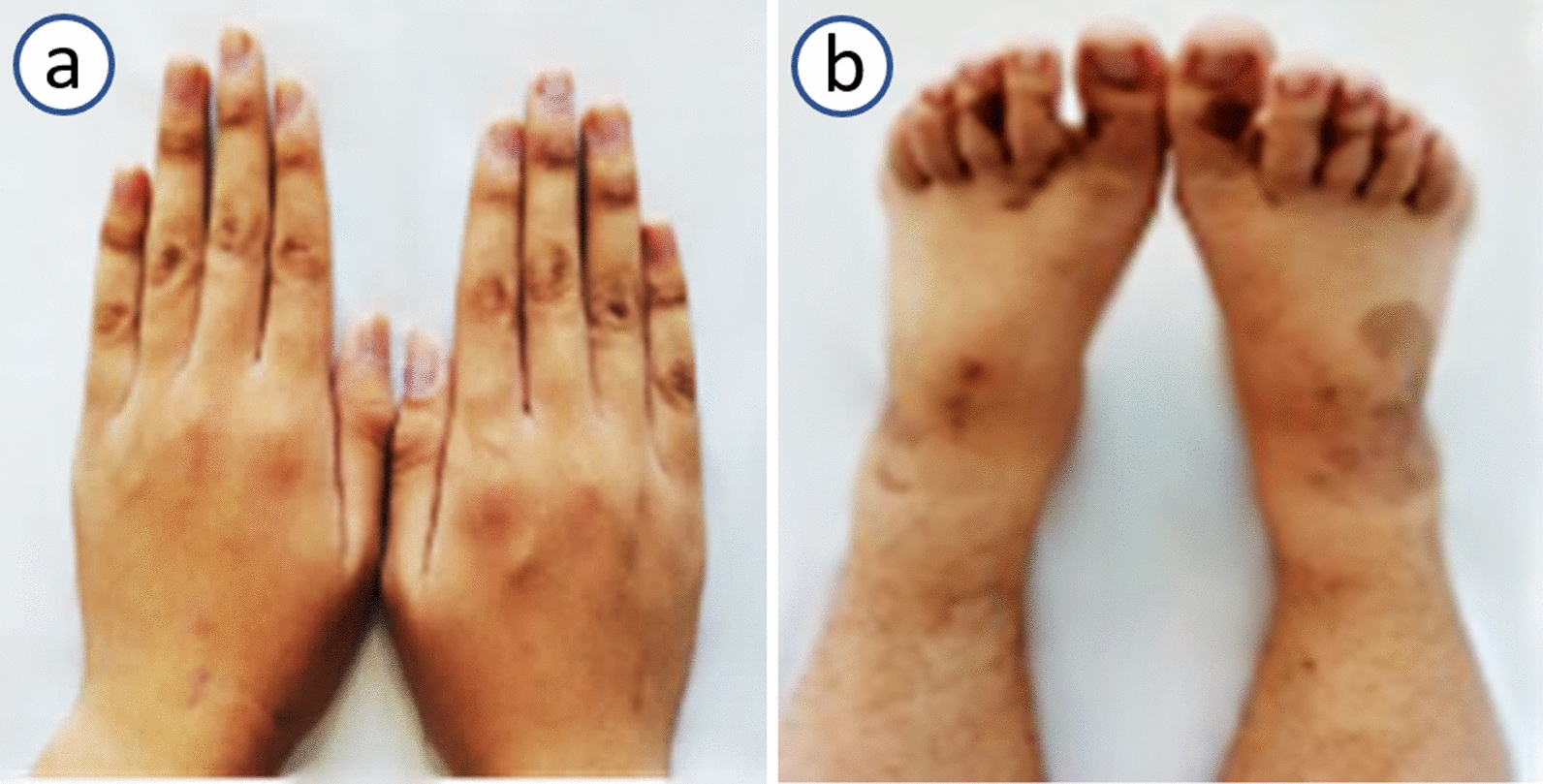
Mesh pigmentation on the exposed areas of the skin on both hands (**a**) and feet (**b**)

A 16-year-old Han male patient experienced unexplained black stool for 7 days, accompanied by dizziness and fatigue. The patient denied any history of alcohol, tobacco, and illicit drug use. His parents were healthy, and there was no history of diseases in the family. On examination, the patient was of normal build and pale. However, there were mesh pigmentations on the exposed areas of the skin on both hands and feet (Fig. 1a, b).

**Fig. 2:**
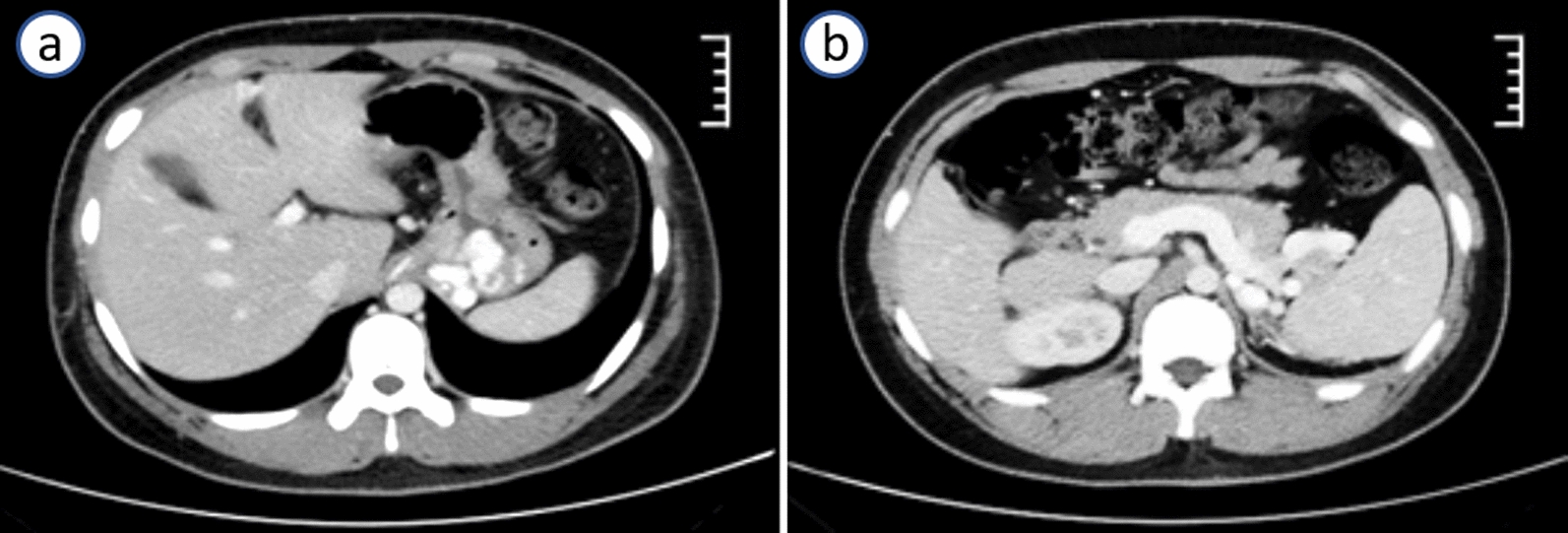
Enhanced computed tomography scan of the abdomen showing varicose veins in the lower esophagus and gastric fundus (**a**), dilation of main portal vein, and splenomegaly (**b**)

**Fig. 3:**
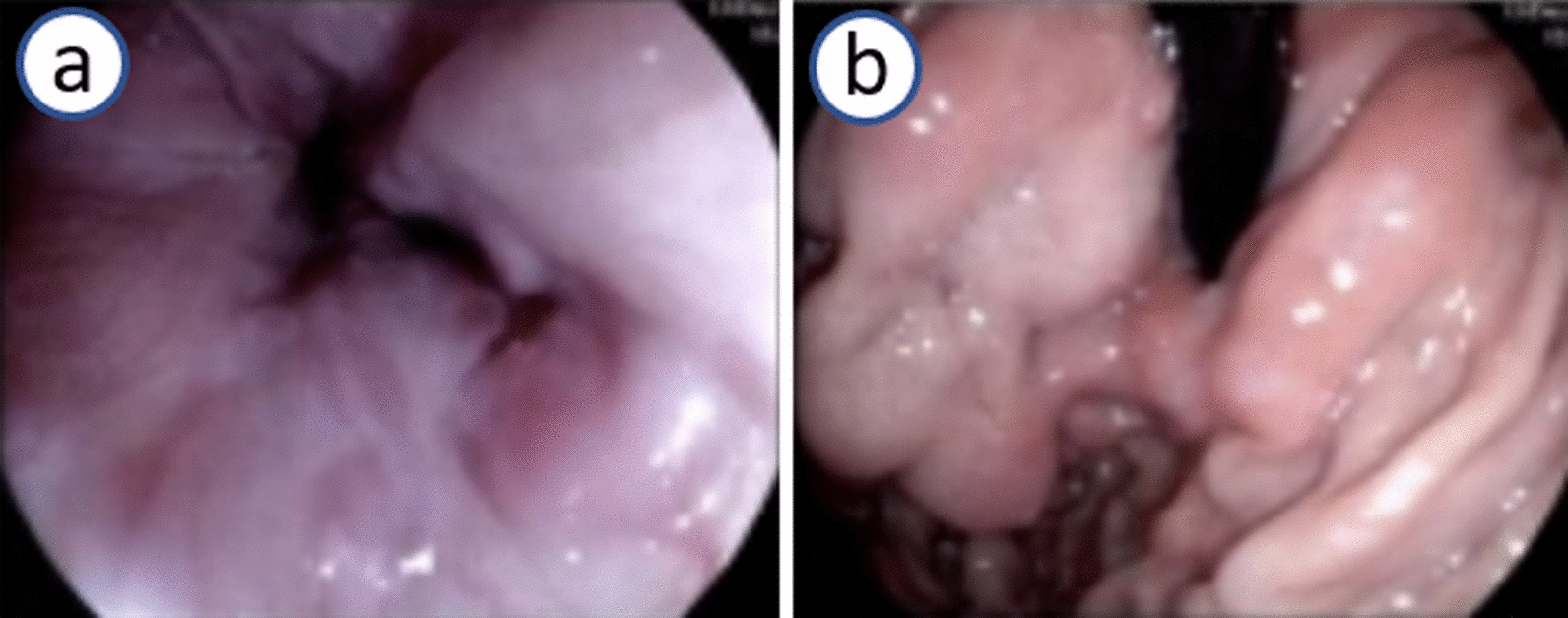
Gastroscopy revealing severe varicose veins in the lower esophagus (**a**) and gastric fundus (**b**)

Routine blood tests on admission displayed reduced red blood cell (RBC) count (2.37 × 10^12^/L) and hemoglobin level (6.9 g/dL). Serum biochemical data were as follows: alanine aminotransferase (ALT) 46.8 U/L, aspartate aminotransferase (AST) 41.3 U/L, γ-glutamyl transpeptidase (GGT) 84.5 U/L, creatinine 59 μM/L, and serum ceruloplasmin 0.326 g/L. The tests for anti-human immunodeficiency virus (HIV), anti-hepatitis C virus (HCV), anti-Epstein–Barr virus (EBV) immunoglobulin M (IgM), anti-cytomegalovirus (CMV) IgM, hepatitis B surface antigen (HBsAg), and HCV RNA were negative. Antinuclear antibodies were negative. A computed tomography (CT) scan of the abdomen revealed multiple varicose veins in the lower esophagus and gastric fundus (Fig. 2), widening of the main vein (diameter of main portal vein of 16.5 cm), and splenomegaly (spleen thickness of 5.5 cm). The liver stiffness measurement (LSM) was measured by FibroScan. The value of LSM was 6.0 kPa. Hepatic artery angiography showed that the main branches of the hepatic artery were well distributed, naturally running from coarse to fine, and with smooth edges. Gastroscopy showed severe varices in the lower esophagus and cardia (Fig. 3a, b).

**Fig. 4:**
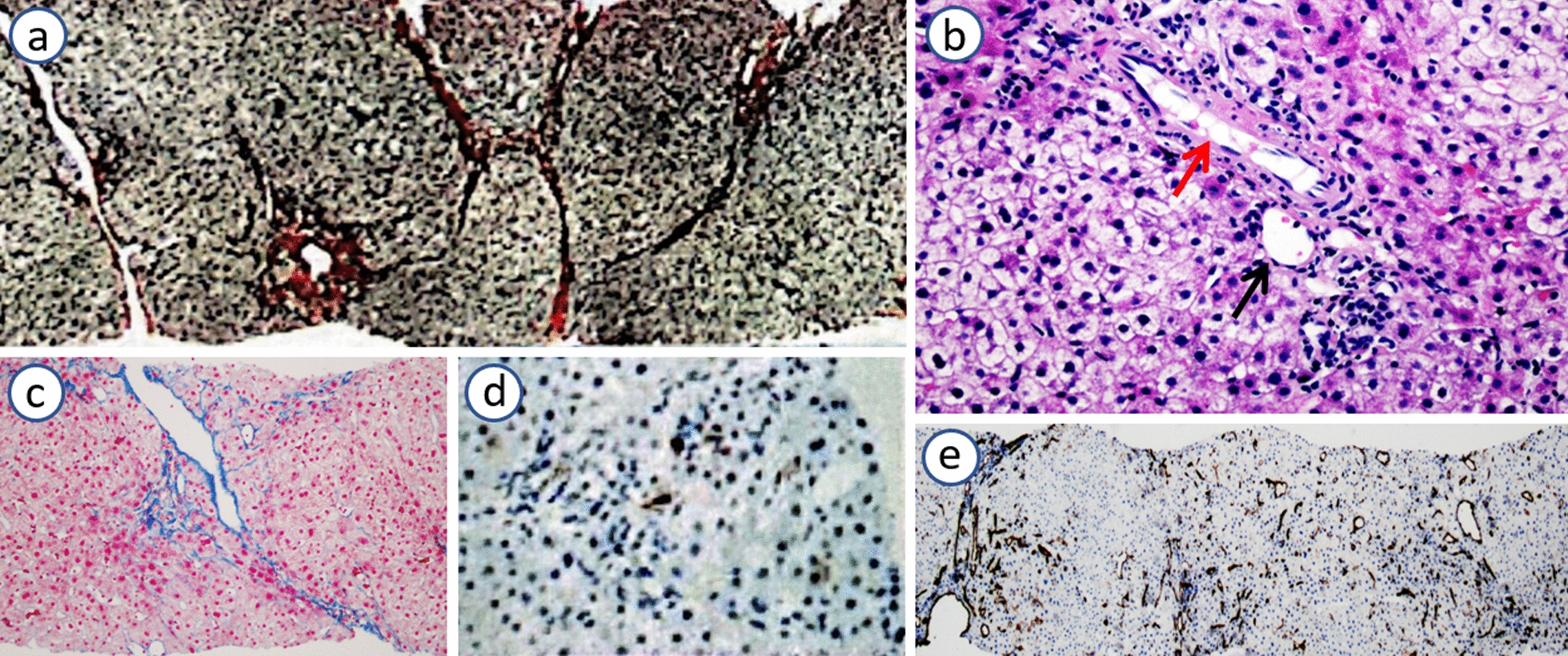
Histological findings of liver biopsy: **a** Reticulin stain showing the regenerative nodularity of the parenchyma (200×); **b** H&E stain showing one hepatic artery (red arrow) with a diameter larger than the parallel portal vein (black arrow) (400×); **c** Masson stain showing perisinusoidal fibrosis and incomplete septa connected to the portal area (200×); **d** immunohistochemistry revealing a few CK7-positive hepatocytes around some portal areas (200×) and (**e**) CD34 positivity in extensive sinusoidal endothelial cells (200×)

The patient underwent a liver biopsy for a possible diagnosis of PSVD. Reticulin staining of the liver biopsy revealed nodular regenerative hyperplasia (Fig. 4a) and Masson staining showed perisinusoidal fibrosis and incomplete septa connected to the portal area (Fig. 4c), suggesting mild ischemic liver fibrosis and no evidence of cirrhotic change of the liver. Hematoxylin and eosin (H&E) staining showed dilation of one hepatic artery in the portal area (Fig. 4b). Immunohistochemical stains revealed a few CK7-positive hepatocytes around some portal areas (Fig. 4d) and CD34 positivity in extensive sinusoidal endothelial cells (Fig. 4e). These results met the diagnostic criteria for PSVD.

**Fig. 5:**
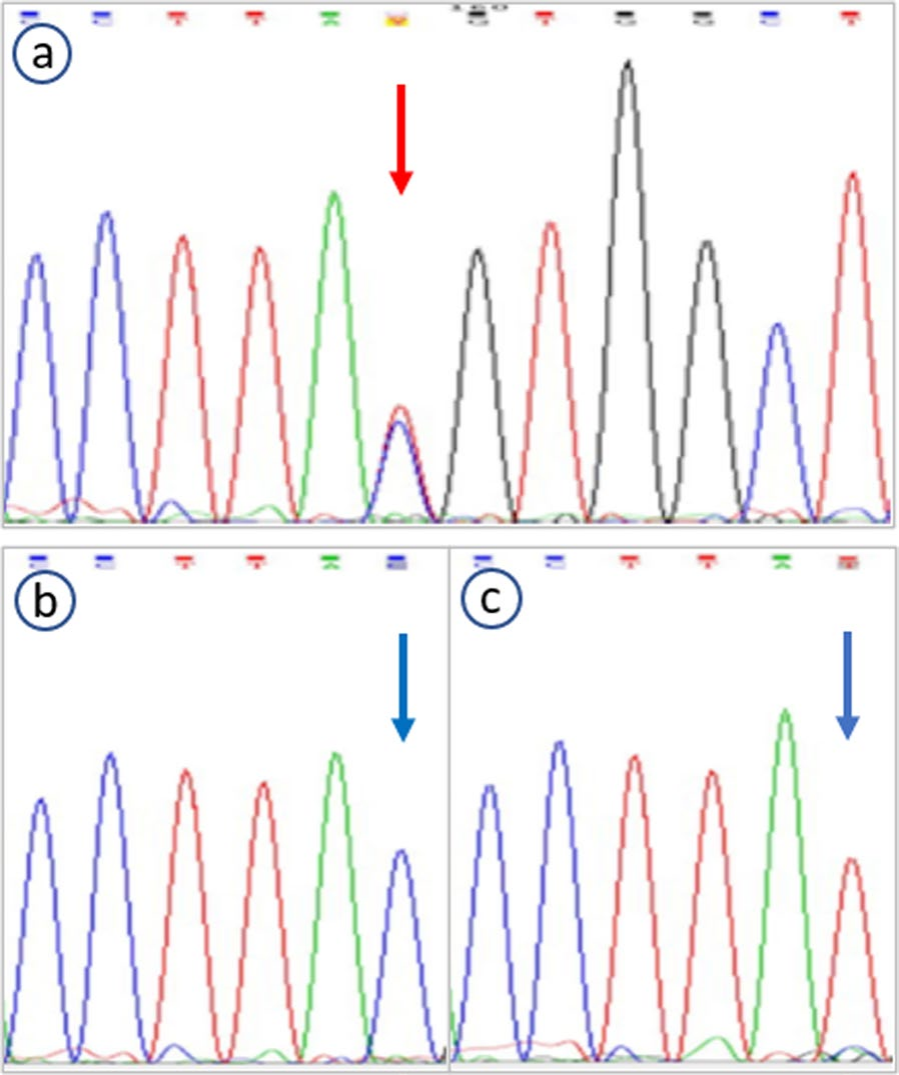
Electropherogram showing heterozygosity for the c.2286 + 1G > A mutation in the *TERT* gene of the patient, occurring de novo (red arrow) (**a**). This mutation is not seen in his father (**b**) or mother (**c**) (blue arrows)

To explore the causes of PSVD and skin pigmentation, medical whole exome sequencing was completed. A heterozygous variation (c.2286 + 1G > A) in the *TERT* gene of the patient occurred de novo (Fig. 5a), as this mutation was not detected in his parents (Fig. 5b, c). To clarify the impact of this *TERT* de novo mutation on telomeres, measurement of telomere length using T/S ratio was completed by quantitative polymerase chain reaction (PCR). The length of his telomeres was less than the first percentile for age.

The patient was diagnosed with *TERT* de novo mutation-related DC and PSVD. He underwent esophageal and gastric variceal ligation treatment. He was then discharged after 7 days and received a carvedilol tablet (12.5 mg) every morning to reduce portal pressure and prevent variceal bleeding. The patient had no dizziness, palpitations, or abdominal discomfort during the carvedilol treatment period. After 6 months, reexamination revealed RBC count of 4.28 × 10^12^/L and hemoglobin level of 7.9 g/dL. He had moderate iron deficiency anemia and started receiving polysaccharide iron complex therapy.

## Discussion

Dyskeratosis congenita and related telomere biology disorders (DC/TBD) are caused by damaged telomere maintenance leading to telomere shortening. The clinical manifestations of DC are highly heterogeneous. Classic DC is characterized by a triad of leukoplakia, nail dystrophy, and reticular pigmentation of the upper chest and neck and/or abnormal pigmentation in other areas of the skin [18]. However, this does not occur in all individuals, owing to variable rates and ages. The diagnosis of DC is made on the basis of the presence of at least two features of the DC clinical triad or one feature of the triad plus bone marrow failure [19] and/or very short telomeres (less than the first percentile for age) in peripheral blood lymphocyte subsets [20], and confirmed by identification of germline pathogenic variants in DC/TBD genes [2, 17]. In the case reported herein, the patient presented mesh pigmentations of the skin on both hands and feet, very short telomeres, and a likely pathogenic de novo variant in the *TERT* gene, thus fully meeting the DC/TBD diagnostic criteria.

The new diagnostic criteria of PSVD can be defined with an adequate liver biopsy on the basis of the presence of at least one of the following three features and the absence of cirrhosis: (*i*.) at least one specific sign of portal hypertension (gastroesophageal varices, porto-systemic collaterals, or portal hypertensive bleeding); (*ii*.) at least one specific histological sign of PSVD (obliterative portal venopathy, nodular regenerative hyperplasia, incomplete septal fibrosis or cirrhosis); and (*iii*.) at least one nonspecific sign of portal hypertension (ascites, splenomegaly, or thrombocytopenia) and at least one nonspecific histological sign of PSVD (portal tract abnormalities: multiplication, dilation of arteries, periportal vascular channels, and aberrant vessels; mild perisinusoidal fibrosis; architectural disturbance; or non-zonal sinusoidal dilation) [5, 6, 21]. In this study, the patient presented esophageal and gastric variceal bleeding owing to portal hypertension and specific histological signs of PSVD, including nodular regenerative hyperplasia, perisinusoidal fibrosis, and incomplete septa fibrosis, as well as the absence of cirrhosis. Thus, the patient fully met the diagnostic criteria for PSVD.

The prevalence of liver disease in patients with DC is approximately 5–10%. The spectrum of hepatic involvement in patients with DC/TBD presents diversity such as cholestasis, liver fibrosis, cirrhosis, and nodular regenerative hyperplasia leading to non-cirrhotic portal hypertension depending on the mode of disease inheritance and gene mutation [22, 23]. A heterogeneous loss-of-function TERT K570N or TERT S368F mutation in two large families, through autosomal dominant inheritance, was previously reported that presented a range of hematologic manifestations from macrocytosis to acute myeloid leukemia and severe liver disease. These *TERT*-related liver diseases were marked by fibrosis, cirrhosis, and non-cirrhotic portal hypertension, the latter of which was featured by nodular regenerative hyperplasia, incomplete septal fibrosis, and CD34-positive sinusoidal endothelial cells, indicating an abnormal proportion of arterial blood flow to the sinuses [22, 24]. A novel *TERT* c.2062 C > G mutation, encoding the Glu668Asp variant, was also identified in the neoplastic tissue of a patient with hepatocellular carcinoma, who had idiopathic familial pulmonary fibrosis and familial cryptogenic cirrhosis [25]. A *TERT* c.2286 + 1G/A mutation in a splicing consensus site was identified in a patient with pulmonary fibrosis, which was estimated as being probably damaging for TERT enzymatic activity, resulting in telomere shortening [13].

In this study, we report a case of a *TERT* de novo c.2286 + G/A mutation associated with DC/TBD and PSVD, which has never been reported before. This mutation in a splicing consensus site might directly interfere with TERT enzymatic activity leading to reduced telomere length and DC/TBD. DC/TBD is characterized by premature aging in different organs, and the potential susceptibility of sinusoidal endothelial cells in the pathological process of cellular aging leads to dysfunction and tension imbalance within itself [26–28]. PSVD is believed to be associated with hepatic and sinusoidal dysfunction, leading to elevated hepatic vascular resistance and increased portal pressure [27, 28]. Imbalances between portal and arterial blood flow leads to incomplete septa fibrosis and nodular regenerative hyperplasia of the liver. The expression of CD34 in sinusoidal endothelial cells was detected in two *TERT* mutation patients with non-cirrhotic portal hypertension, indicating an abnormal proportion of arterial blood flow to the sinuses [22]. Therefore, we speculate that the *TERT* de novo c.2286 + 1G/A mutation can directly interfere with TERT enzymatic activity, resulting in telomere shortening, dyskeratosis congenita, and PSVD. The pathophysiological changes of PSVD are dysfunction of sinusoidal endothelial cells, imbalance of hepatic portal and arterial blood flow, and an increased resistance of the intrahepatic portal vein. This case is reported for the first time and its prognostic features are difficult to predict. Therefore, regular monitoring of portal hypertension and timely resolution of potential bleeding risks from varicose veins are necessary. Additionally, scientific diet and safe medicine for pregnant women, as well as specialized medical institutions, are recommended for the prevention and care of such genetic diseases [29, 30].

## Conclusion

We report a rare case of *TERT* de novo c.2286 + 1G/A mutation-associated DC/TBD with PSVD. When discovering reticular rash, cryptogenic liver disease, or portal hypertension, it is necessary to perform whole exome sequencing and chromosome length testing to clarify the possibility of this disease. In the future, it will be necessary to establish an animal model with the same site deletion to verify the reasons for the vulnerability of sinusoidal endothelial cells and exclude the possibility of other triggering factors.

## Data Availability

Supporting data related to this case report can be made available to the corresponding author upon reasonable request, subject to patient privacy considerations.
